# Reply to McDonald, “Protections against the Risk of Airborne SARS-CoV-2 Infection”

**DOI:** 10.1128/mSystems.00435-20

**Published:** 2020-06-16

**Authors:** Leslie Dietz, Patrick F. Horve, David A. Coil, Mark Fretz, Jonathan A. Eisen, Kevin Van Den Wymelenberg

**Affiliations:** a Biology and the Built Environment Center, University of Oregon, Eugene, Oregon, USA; b Genome Center, University of California—Davis, Davis, California, USA; c Institute for Health in the Built Environment, University of Oregon, Portland, Oregon, USA; d Department of Evolution and Ecology, University of California—Davis, Davis, California, USA; e Department of Medical Microbiology and Immunology, University of California—Davis, Davis, California, USA; f Genome Center, University of California—Davis, Davis, California, USA

## REPLY

We thank Dr. McDonald (1) for his close reading of our paper (2) and acknowledge that he makes important arguments for exercising precautions in order to prevent built environment-mediated transmission of SARS-CoV-2. We would like to address two specific points that Dr. McDonald brings up in relation to our original publication. Dr. McDonald states that we questioned the effectiveness [“Given the virus’s small size, Dietz et al. (3) and others have questioned whether N95 respirators block them.”] of N95 respirators at protecting the wearer from particles “larger than 3 μm.” First, throughout our paper, we do not address the efficiency or efficacy of N95 respirators. We wrote “as masks become available, and while prioritizing access to masks for health care workers that are in a higher risk environment daily, wearing a mask would be prudent,” as was recommended by the Centers for Disease Control (3) and the Food and Drug Administration (4). Since the time of publication, views on the use of cloth and surgical masks in public have changed (https://www.cdc.gov/coronavirus/2019-ncov/prevent-getting-sick/diy-cloth-face-coverings.html), and we acknowledge that they can provide some protection against SARS-CoV-2 but should be combined with other infection prevention measures, such as physical distancing and handwashing.

In regard to Dr. McDonald’s comments about virus size and whether it will be filtered by N95 respirators, we would like to point out that we issued a correction of the paper accepting that what is important here is the size of particles containing the virus and not the size of the virus itself.

Second, we would like to address Dr. McDonald’s claim that installing HEPA filters in homes will “eliminate the risk of air duct transmission.” HEPA filters are very good at removing particles in a broad spectrum of sizes from the air. However, no filter or mechanical system is perfect (5), as demonstrated by recent events at a Children’s Hospital in Seattle, WA (6). Furthermore, the majority of existing built environment mechanical ventilation systems are not capable of having a HEPA filter or high-level MERV filter installed without retrofit measures and consultation with mechanical engineers and mechanical contractors (7).

We thank Dr. McDonald for assessing our review so critically. We do believe, however, that the current body of knowledge supports our statements.
